# WGVD: an integrated web-database for wheat genome variation and selective signatures

**DOI:** 10.1093/database/baaa090

**Published:** 2020-11-11

**Authors:** Jierong Wang, Weiwei Fu, Rui Wang, Dexiang Hu, Hong Cheng, Jing Zhao, Yu Jiang, Zhensheng Kang

**Affiliations:** State Key Laboratory of Crop Stress Biology for Arid Areas and College of Plant Protection, Northwest A&F University, 3 Taicheng Rd, Yangling 712100, Shaanxi, China; College of Animal Science and Technology, Northwest A&F University, 22 Xinong Rd, Yangling, 712100, Shaanxi, China; College of Animal Science and Technology, Northwest A&F University, 22 Xinong Rd, Yangling, 712100, Shaanxi, China; College of Animal Science and Technology, Northwest A&F University, 22 Xinong Rd, Yangling, 712100, Shaanxi, China; College of Animal Science and Technology, Northwest A&F University, 22 Xinong Rd, Yangling, 712100, Shaanxi, China; State Key Laboratory of Crop Stress Biology for Arid Areas and College of Plant Protection, Northwest A&F University, 3 Taicheng Rd, Yangling 712100, Shaanxi, China; College of Animal Science and Technology, Northwest A&F University, 22 Xinong Rd, Yangling, 712100, Shaanxi, China; State Key Laboratory of Crop Stress Biology for Arid Areas and College of Plant Protection, Northwest A&F University, 3 Taicheng Rd, Yangling 712100, Shaanxi, China

## Abstract

Bread wheat is one of the most important crops worldwide. With the release of the complete wheat reference genome and the development of next-generation sequencing technology, a mass of genomic data from bread wheat and its progenitors has been yield and has provided genomic resources for wheat genetics research. To conveniently and effectively access and use these data, we established Wheat Genome Variation Database, an integrated web-database including genomic variations from whole-genome resequencing and exome-capture data for bread wheat and its progenitors, as well as selective signatures during the process of wheat domestication and improvement. In this version, WGVD contains 7 346 814 single nucleotide polymorphisms (SNPs) and 1 044 400 indels focusing on genic regions and upstream or downstream regions. We provide allele frequency distribution patterns of these variations for 5 ploidy wheat groups or 17 worldwide bread wheat groups, the annotation of the variant types and the genotypes of all individuals for 2 versions of bread wheat reference genome (IWGSC RefSeq v1.0 and IWGSC RefSeq v2.0). Selective footprints for *Aegilops tauschii*, wild emmer, domesticated emmer, bread wheat landrace and bread wheat variety are evaluated with two statistical tests (*F*_ST_ and Pi) based on SNPs from whole-genome resequencing data. In addition, we provide the Genome Browser to visualize the genomic variations, the selective footprints, the genotype patterns and the read coverage depth, and the alignment tool Blast to search the homologous regions between sequences. All of these features of WGVD will promote wheat functional studies and wheat breeding.

**Database URL:**

http://animal.nwsuaf.edu.cn/code/index.php/Wheat

Bread wheat (*Triticum aestivum*) is the most widely grown food crop in the world and provides the major source of the calories and protein humans consumed. It has an allohexaploid genome that includes three subgenomes (AABBDD) (1). Its origin is ascribed to two evolutionary events; first, the formation of tetraploid domesticated emmer (*Triticum dicoccum*, AABB) from domesticating tetraploid wild emmer (*Triticum dicoccoides,* AABB), and second, the evolution of hexaploid bread wheat by hybridization of tetraploid domesticated emmer with diploid *Aegilops tauschii* (*Ae. tauschii*, DD) (2, 3). During the process of polyploidization, domestication, improvement and dissemination, wheat underwent natural and artificial selection for yield, quality and adapting the different eco-geographic habitats (4–7), which led to the formation of diverse populations. The release of the complete bread wheat reference genome (8) and the decreasing costs of sequencing have resulted in rapidly accumulating genome variation data and analyzing selective signatures. As one of the most important types of molecular variation, single nucleotide polymorphism (SNP) is broadly distributed in the genome, which makes it a powerful marker for wheat population genetics study, particularly for the selection regions analysis, and the gene loci identification associated with desired traits (9, 10). Therefore, establishing a comprehensive web-database, which integrates the genomic variations and displays the selective footprints from populations, is vital for wheat genomics research.

Here, we established a database WGVD containing the genome variations and the selective signatures focusing on wheat domestication and improvement from five groups: wild emmer, domesticated emmer, *Ae. tauschii*, bread wheat landrace and bread wheat variety. In the current version, we have collected high-resolution whole-genome resequencing data and exome sequencing data from 968 bread wheat and its progenitors (11–13). A total of 7 346 814 SNPs and 1 044 400 indels concentrating on genic regions and upstream or downstream regions have been included in WGVD. Furthermore, we have implemented two statistical methods, nucleotide diversity (Pi) (14) and Weir and Cockerham’s *F*_ST_ (15), to calculate the selective scores based on the 93 whole-genome resequencing wheat accessions. WGVD would be a valuable platform for the global research community to promote the wheat-related studies.

## Database structure and content

**Figure 1.**
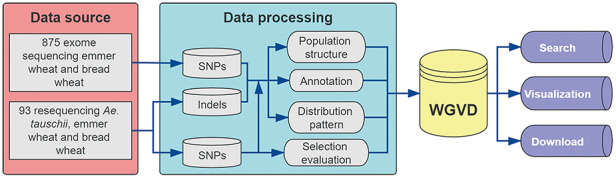
Data sources and analysis pipeline used to build the database.

The WGVD contains SNPs, indels, signals of selection, genome browser and alignment tool (Blast) for wheat. Figure 1 shows the analysis pipeline used to build the database. The detailed description is provided in the following sections.

## Sample information

**Figure 2.**
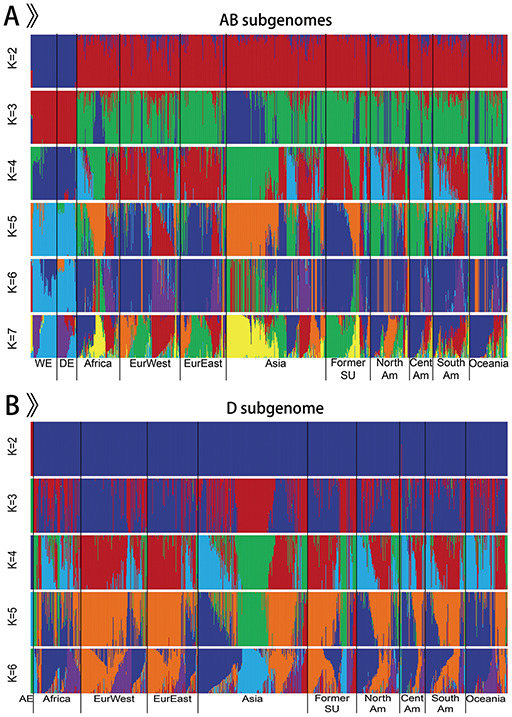
Population structure of 968 accessions.

Our database brings together the published wheat genome sequence data from 1 whole-genome resequencing dataset of 93 wheat accessions including 5 *Ae. tauschii*, 20 wild emmer, 5 domesticated emmer, 29 bread wheat landraces and 34 bread wheat varieties, and two exome sequencing datasets of 875 wheat accessions including 33 wild emmer, 35 domesticated emmer and 807 bread wheat (11, 12) (Figure 2). Overall, genomic information from 968 bread wheat and its progenitors comprising 5 diploid *Ae. tauschii* (AE), 53 tetraploid wild emmer wheat (WE), 40 tetraploid domesticated emmer wheat (DE) and 870 hexaploid bread wheat is obtained and analyzed. Bread wheat contains 315 landraces and 471 varieties. According to the geographic origin, tetraploid wild emmer wheat is separated into three groups: Southern Levant 1 (WE Sorth1), Southern Levant 2 (WE Sorth2) and Northern Levant (WE North), including 7, 30 and 16 samples, respectively. Likewise, except for 7 accessions of unknown geographic origin, hexaploid bread wheat contains 9 groups: 87 African wheat (Africa), 122 Western European wheat (EurWest), 93 Eastern European wheat (EurEast), 201 Asian wheat (Asia), 90 wheat from Former Soviet Union (Former SU), 79 North American wheat (NorthAm), 74 South American wheat (SouthAm), 47 Central American wheat (CentAm) and 70 Oceanian wheat (Oceania). Geographical origins and other detailed information for all samples can be obtained from the homepage and ‘Sample Table’ page in WGVD.

A. Population structure of 963 emmer wheat and bread wheat accessions on AB subgenomes. B. Population structure of 875 *Ae. tauschii* and bread wheat accessions on D subgenome.

## SNPs and indels information

In addition to the SNP and indel dataset generated by Cheng *et al.* (13), we have downloaded the published SNP dataset from two other studies (11, 12). Due to the different reference genomes used for calling SNPs from some emmer wheat (11), we selected the 100 bp flanking sequences of each SNP in the reference genome of emmer to align onto the bread wheat reference genome IWGSC RefSeq v1.0 using BLAST. The parameters, alignment coverage > 50% and identities > 90%, were used for defining a blast hit. The SNPs and indels (1–50 bp) located in the genic regions and the flanking segment (3 kb upstream or downstream regions) were selected from the 93 resequencing data (13). Finally, we integrated all the processed SNPs and indels, and obtained a total of 7 346 814 SNPs and 1 044 400 indels. PLINK (16) was used to calculate minor allele frequency (MAF) for all wheat and allele frequencies for each ploidy wheat group and the worldwide bread wheat group. The snpEff (version 4.3p) software was used to annotate variant effects of SNPs and indels (17). Given that two versions of bread wheat genome (IWGSC RefSeq v1.0 and IWGSC RefSeq v2.0), we have converted each SNP and indel on IWGSC RefSeq v1.0 by aligning the flanking sequences onto IWGSC RefSeq v2.0 using BLAST with the parameters ‘alignment coverage > 99% and identities > 99%’.

**Table 1.** Statistical terms for selective signatures in the WGVD.

Statistical term	Abbreviation	Population 1	Population 2	Windows
Nucleotide diversity	Pi	*Ae. tauschii* (AE)		100k
Cockerham & Weir Fst	*F*_ST_	Wild emmer (WE)	Other four groups	100k
		Domesticated emmer (DE)		
		Landrace		
		Variety		

## Population structure

We only retained the integrated SNPs with a maximum missing rate < 0.9 for population structure analysis. The software ADMIXTURE (version 1.3.0) was used to analyze the population structure of all accessions (18). ADMIXTURE was run from *K *= 2 to *K *= 7 with 20 bootstrap replicates on AB subgenomes of 963 individuals (Figure 2A) and from *K *= 2 to *K *= 6 with 20 bootstrap replicates on D subgenome of 875 individuals (Figure 2B). The results showed that the separation of emmer wheat and *Ae. tauschii* from bread wheat, as well as the high-level interpopulation admixture of bread wheat, which were consistent with previous findings (4, 12, 13, 19). To better illustrate the population structure of hexaploid bread wheat and tetraploid emmer wheat, and to reduce the influence of high repeat of bread wheat genome, particularly in the intergenic regions, we selected the SNPs in the genic regions to construct NJ trees with PHYLIP (version 3.68) (20) for 870 bread wheat and 93 emmer wheat, respectively. These trees were visualized with Interactive Tree of Life (21). Except for the difference caused by the sequencing methods, the result was in accordance with previous depictions (12, 13).

## Selection evaluation

WGVD provides selective signatures from five groups including *Ae. tauschii*, wild emmer, domesticated emmer, bread wheat landrace and bread wheat variety. Due to the higher density and broader distribution of SNPs from the whole-genome resequencing data than from the exome sequencing data, we selected the whole-genome SNPs from 93 wheat accessions to evaluate selective footprints with Cockerham and Weir Fst (*F*_ST_), and nucleotide diversity (Pi) (13) (Table 1).

## Database implementation

The WGVD was built with Apache, PHP, MySQL, JavaScript and HTML. The processing of variations (SNPs/indels) and selection scores were processed with Perl scripts. And the corresponding data were deposited in the MySQL database. The implementation of data search, visualization and download were used with HTML5 and JavaScript. Moreover, ViroBLAST and the UCSC Genome Browser (Gbrowse) were introduced into the WGVD (22, 23).

## Usage

WGVD can be accessed through a user-friendly interface. We provide the ‘Manual’ in ‘Documentation’ page for users to access the database. Generally, WGVD has four main functionalities: variation search, genomic selection search, genome browser and alignment search tool (BLAST).

## Variation search

**Figure 3.**
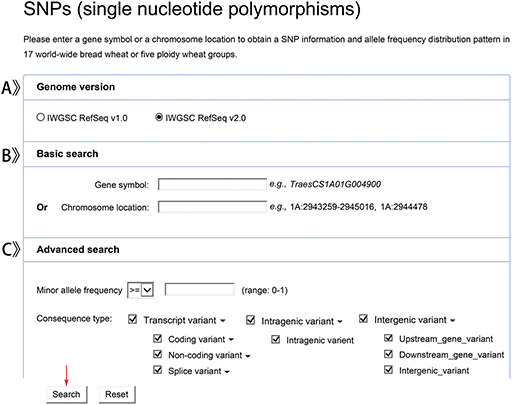
Screenshots of a SNP data search.

We provide two versions of bread wheat genome (IWGSC RefSeq v1.0 and IWGSC RefSeq v2.0) for users to choose (Figure 3A). Users can get detailed SNPs or indels information by a given gene name or genomic location in the reference genome (Figure 3B). In addition, users can set the parameters, MAF and consequence type to search for variations of interest in a more efficient and faster manner (Figure 3C).

A. Genome version items. B. Basic search items. C. Advanced search items.

**Figure 4.**
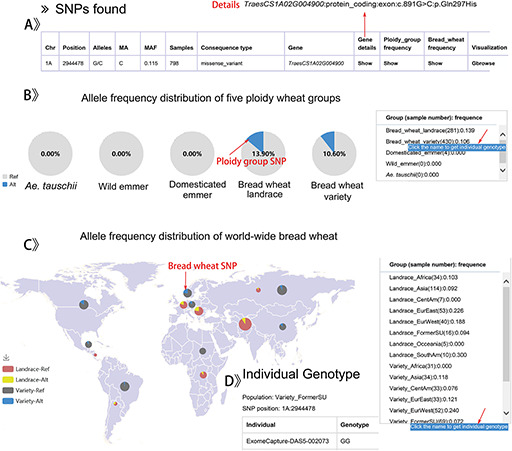
The result display by searching a SNP data for example.

The retrieved results are shown with an interactive table and graph. Users can get details of SNPs and indels in 5 ploidy wheat groups or 17 worldwide bread wheat groups (Figure 4A, B, C), such as the variant position, allele, MAF, variant type and the distribution pattern of allele frequency. By clicking the group name, users can also obtain the information on individual genotypes (Figure 4D).

A. Detailed annotation of a SNP in *TraesCS1A02G004900* gene. B. The pie-chart of allele frequency distribution for five ploidy wheat groups. C. Display format of the allele frequency for the SNP of 17 worldwide bread wheat groups. D. The individual genotypes in the group of Variety_FormerSU.

## Genomic selection search

**Figure 5.**
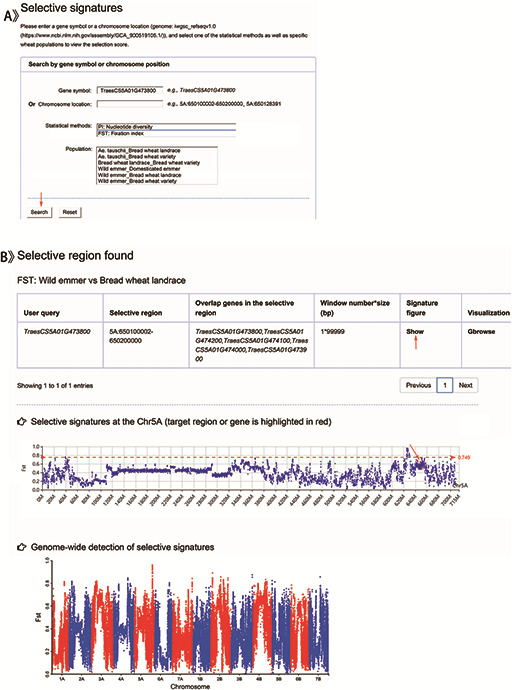
Screenshots of a search for genomic selection data and the result display for example.

Users can get the selective scores by choosing a given gene name or genomic location, one of the statistical methods (Pi or *F*_ST_) and one of the wheat groups (*Ae. tauschii*, wild emmer, domesticated emmer, bread wheat landrace or bread wheat variety) (Table 1 and Figure 5A). Table 1 displays the results including a selective region and the corresponding overlapping genes (Figure 5B). While clicking the ‘show’ button, users can obtain the selective signatures showing with Manhattan plot and the common graphic, in which the target gene or region is highlighted with red color. To prove the function of our database, we retrieved *TraesCS5A01G473800* (Figure 5A) identified as gene *Q* controlling free threshing (24) by BLASTn. The result displayed a significant selection signal between wild emmer and bread wheat landrace (Figure 5B), which accorded with previous reports (12, 13, 24).

A. Search items including gene symbol, chromosome location, one of the statistical tests (Pi and *F*_ST_) and specific ploidy wheat groups. B. Detailed annotation for the target gene or region in the variant grid and the corresponding selective signatures at the chromosome and whole-genome levels, respectively. An example of selective signal *Fst* of *TraesS5A01G473800* gene between wild emmer and bread wheat landrace.

## Genome browser

**Figure 6.**
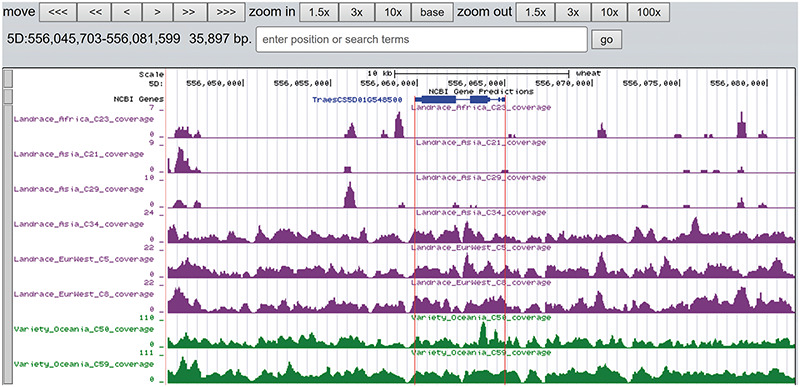
The read coverage depth on the region of gene *TraesCS5D01G548500* in eight bread wheat accessions. The gene is absent in three landrace (C21, C23 and C29) as indicated by the few reads alignment, while it is present in the other five accessions.

**Figure 7.**
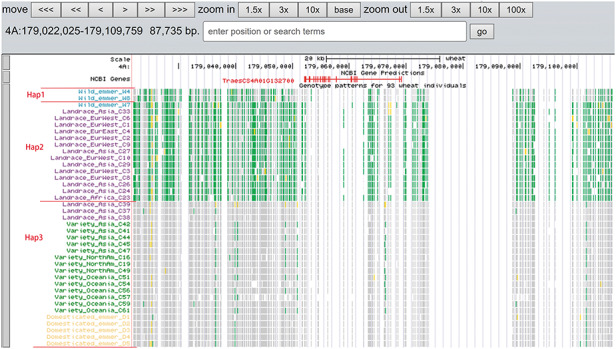
Three haplotypes (Hap1, Hap2 and Hap3) for gene *TraesCS4A01G132700* region among wild emmer, domesticated emmer, landrace and variety based on the genotype pattern with homozygous reference in gray, heterozygous variant in yellow and homozygous variant in green.

**Figure 8.**
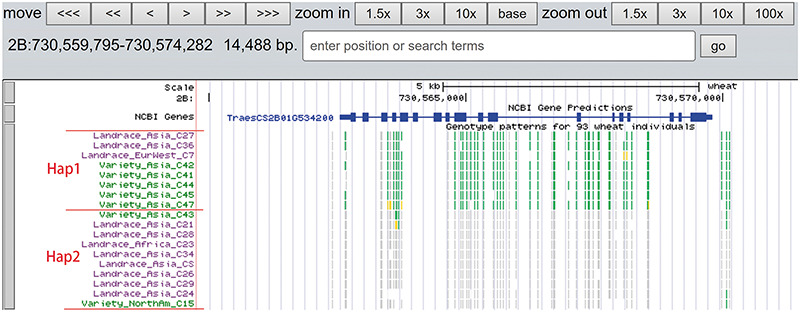
Two haplotypes (Hap1 and Hap2) for gene *TraesCS2B01G534200* region among bread wheat based on the genotype pattern with homozygous reference in gray, heterozygous variant in yellow and homozygous variant in green.

**Figure 9.**
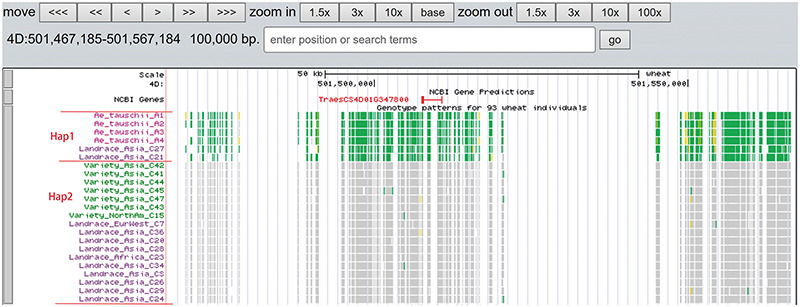
Two haplotypes (Hap1 and Hap2) for gene *TraesCS4D01G347800* region among *Ae. tauschii*, landrace and variety based on the genotype pattern with homozygous reference in gray, heterozygous variant in yellow and homozygous variant in green.

A total of 19 and 6 tracks have been provided for the reference genome IWGSC RefSeq v1.0 and IWGSC RefSeq v2.0, respectively. By retrieving a special gene name or chromosome location, users can view SNPs, indels and signals of selection as a whole. Due to the large size of whole-genome resequencing BAM files (e.g. ∼50 GB for each file), the read coverage depth of the genome was computed using bamCoverage from deepTools2 (25) with the option ‘--binSize 100’ and shown in ‘Reads coverage’ track, which helps to visualize the distribution of genes for each accession. For example, *TraesCS5D01G548500*, the homologous gene of disease resistance protein RPP13 in *Brachypodium distachyon*, is absent in landrace C21, C23 and C29 as indicated by the few reads alignment (Figure 6). Most notably, the genotype patterns of 93 wheat accessions are shown in ‘Genotype patterns’ track with homozygous reference in gray, heterozygous variant in yellow and homozygous variant in green, which would allow users to observe haplotype blocks and different haplotypes. Figure 7 displays a region on chromosome 4A including *TraesCS4A01G132700* identified as *ABCT* gene (26). The genotype pattern in this region obviously displays 3 distinct haplotypes, in which wild emmer W7 shares the same haplotype with 14 landrace accessions. Gene *TraesCS2B01G534200* annotated as being an NADPH-cytochrome P450 reductase, which co-localized with the QTLs related with resistance to leaf rust (27), shows two different haplotypes among bread wheat (Figure 8). Gene *TraesCS4D01G347800* is the homologous gene of *FPF1* in *Arabidopsis thaliana* controlling flower development, in which Asian landrace C21, C27 and four *Ae. tauschii* accessions are grouped into one haplotype (Figure 9).

## Alignment search tool (BLAST)

Users can input sequences in fasta format to search the homologous regions in the reference genome IWGSC RefSeq v1.0 and IWGSC RefSeq v2.0.

## Discussion

WGVD is an open-source web-based database aggregating the SNPs collected from 968 bread wheat and its progenitors, indels, as well as selective signatures during the process of wheat domestication and improvement based on the whole-genome SNPs from 93 wheat individuals. Compared to the previous databases including wheat variations, such as GrainGenes (28), dbSNP (29), Wheat@URGI (30) and Wheat-SnpHub (31), WGVD provides the allele frequency distribution pattern of each SNP or indel in diverse groups based on all collected samples, the individual genotypes, the read coverage depth and the detailed sample information on individuals, which could improve the utilization of data and contribute to the population genetic analysis. Moreover, we provide selection scores for five groups of *Ae. tauschii*, wild emmer, domesticated emmer, bread wheat landrace and bread wheat variety by using two statistical terms This resource facilitates users to identify the selection loci or genes associated with phenotypic changes and study the corresponding mechanisms Users can also download sample list, variation data and selection signal data from WGVD for other studies of interest. We believe that our database facilitates wheat evolutionary studies and functional genes mining.

The database will be updated regularly with new released resequencing data of bread wheat and its progenitors. We will provide uploading functionality for users to submit resequencing data or SNP list directly to WGVD. We will also provide other genetic statistical methods, such as *H*_p_ (32), iHS (33), XP-EHH (34) and XP-CLR (35) and recombination maps to WGVD, as well as selective signatures for wheat of different geographical origins. Furthermore, structural variations of wheat genomes will be integrated into the database.

## References

[1] Pont C., Leroy T., Seidel M. et al. (2019) Tracing the ancestry of modern bread wheats. *Nat. Genet.*, 51, 905–911. 31043760 10.1038/s41588-019-0393-z

[2] Dvorak J., Luo M.-C., Yang Z.-L. et al. (1998) The structure of the *Aegilops tauschii* genepool and the evolution of hexaploid wheat. *Theor. Appl. Genet.*, 97, 657–670.

[3] Luo M.-C., Yang Z.-L., You F.M. et al. (2007) The structure of wild and domesticated emmer wheat populations, gene flow between them, and the site of emmer domestication. *Theor. Appl. Genet.*, 114, 947–959. 17318496 10.1007/s00122-006-0474-0

[4] Cavanagh C.R., Chao S., Wang S. et al. (2013) Genome-wide comparative diversity uncovers multiple targets of selection for improvement in hexaploid wheat landraces and cultivars. *Proc. Natl. Acad. Sci. U. S. A.*, 110, 8057–8062. 23630259 10.1073/pnas.1217133110 PMC3657823

[5] Lopes M.S., El-Basyoni I., Baenziger P.S. et al. (2015) Exploiting genetic diversity from landraces in wheat breeding for adaptation to climate change. *J. Exp. Bot.*, 66, 3477–3486. 25821073 10.1093/jxb/erv122

[6] Zhou Y., Chen Z., Cheng M. et al. (2018) Uncovering the dispersion history, adaptive evolution and selection of wheat in China. *Plant Biotechnol. J.*, 16, 280–291. 28635103 10.1111/pbi.12770 PMC5785339

[7] Liu J., Rasheed A., He Z. et al. (2019) Genome-wide variation patterns between landraces and cultivars uncover divergent selection during modern wheat breeding. *Theor. Appl. Genet.*, 132, 2509–2523. 31139853 10.1007/s00122-019-03367-4

[8] Appels R., Eversole K., Stein N. et al. (2018) Shifting the limits in wheat research and breeding using a fully annotated reference genome. *Sci. (80-.)*, 361, eaar7191. 10.1126/science.aar7191 30115783

[9] Rasheed A., Mujeeb-Kazi A., Ogbonnaya F.C. et al. (2018) Wheat genetic resources in the post-genomics era: promise and challenges. *Ann. Bot.*, 121, 603–616. 29240874 10.1093/aob/mcx148 PMC5852999

[10] Heslot N., Jannink J.-L. and Sorrells M.E. (2015) Perspectives for genomic selection applications and research in plants. *Crop Sci.*, 55, 1–12.

[11] Avni R., Nave M., Barad O. et al. (2017) Wild emmer genome architecture and diversity elucidate wheat evolution and domestication. *Sci. (80-.).*, 357, 93–97. 10.1126/science.aan0032 28684525

[12] He F., Pasam R., Shi F. et al. (2019) Exome sequencing highlights the role of wild-relative introgression in shaping the adaptive landscape of the wheat genome. *Nat. Genet.*, 51, 896–904. 31043759 10.1038/s41588-019-0382-2

[13] Cheng H., Liu J., Wen J. et al. (2019) Frequent intra- and inter-species introgression shapes the landscape of genetic variation in bread wheat. *Genome Biol.*, 20, 1–16. 31300020 10.1186/s13059-019-1744-x PMC6624984

[14] Nei M. and Li W.H. (1979) Mathematical model for studying genetic variation in terms of restriction endonucleases. *Proc. Natl. Acad. Sci. U. S. A.*, 76, 5269–5273. 291943 10.1073/pnas.76.10.5269 PMC413122

[15] Weir B.S. and Cockerham C.C. (1984) Estimating F-statistics for the analysis of population structure. *Evol. (N. Y).*, 38, 1358–1370. 10.1111/j.1558-5646.1984.tb05657.x 28563791

[16] Purcell S., Neale B., Todd-Brown K. et al. (2007) PLINK: a tool set for whole-genome association and population-based linkage analyses. *Am. J. Hum. Genet.*, 81, 559–575. 17701901 10.1086/519795 PMC1950838

[17] Cingolani P., Platts A., Wang L.L. et al. (2012) A program for annotating and predicting the effects of single nucleotide polymorphisms, SnpEff: SNPs in the genome of *Drosophila melanogaster* strain w1118; iso-2; iso-3. *Fly (Austin)*, 6, 80–92. 22728672 10.4161/fly.19695 PMC3679285

[18] Alexander D.H., Novembre J. and Lange K. (2009) Fast model-based estimation of ancestry in unrelated individuals. *Genome Res.*, 19, 1655–1664. 19648217 10.1101/gr.094052.109 PMC2752134

[19] Wang S., Wong D., Forrest K. et al. (2014) Characterization of polyploid wheat genomic diversity using a high-density 90 000 single nucleotide polymorphism array. *Plant Biotechnol. J.*, 12, 787–796. 24646323 10.1111/pbi.12183 PMC4265271

[20] Felsenstein J. (1989) PHYLIP-Phylogenetic Inference Package (Version 3.2). *Cladistics*, 5, 164–166.

[21] Letunic I. and Bork P. (2007) Interactive Tree Of Life (iTOL): an online tool for phylogenetic tree display and annotation. *Bioinf.*, 23, 127–128. 10.1093/bioinformatics/btl529 17050570

[22] Deng W., Nickle D.C., Learn G.H. et al. (2007) ViroBLAST: a stand-alone BLAST web server for flexible queries of multiple databases and user’s datasets. *Bioinf.*, 23, 2334–2336. 10.1093/bioinformatics/btm331 17586542

[23] Casper J., Zweig A.S., Villarreal C. et al. (2018) The UCSC Genome Browser database: 2018 update. *Nucleic Acids Res.*, 46, D762–D769. 29106570 10.1093/nar/gkx1020 PMC5753355

[24] Simons K.J., Fellers J.P., Trick H.N. et al. (2006) Molecular characterization of the major wheat domestication gene Q. *Genetics*, 172, 547–555. 16172507 10.1534/genetics.105.044727 PMC1456182

[25] Ram F., Ryan D.P., Bhardwaj V. et al. (2016) deepTools2: a next generation web server for deep-sequencing data analysis. *Nucleic Acids Res.*, 44, 160–165. 10.1093/nar/gkw257 PMC4987876 27079975

[26] Dvorak J. et al. (2006) Molecular characterization of a diagnostic DNA marker for domesticated tetraploid wheat provides evidence for gene flow from wild tetraploid wheat to hexaploid wheat. *Mol. Biol. Evol.*, 23, 1386–1396. 16675504 10.1093/molbev/msl004

[27] Aoun M., Breiland M., Turner M.K. et al. (2016) Genome-Wide Association Mapping of Leaf Rust Response in a Durum Wheat Worldwide Germplasm Collection. *Plant Genome*, 9, 1–24. 10.3835/plantgenome2016.01.0008 27902791

[28] Matthews D.E. et al. (2003) GrainGenes, the genome database for small-grain crops. *Nucleic Acids Res.*, 31, 183–186. 12519977 10.1093/nar/gkg058 PMC165505

[29] Sherry S.T. (2001) dbSNP: the NCBI database of genetic variation. *Nucleic Acids Res.*, 29, 308–311. 11125122 10.1093/nar/29.1.308 PMC29783

[30] Alaux M., Rogers J., Letellier T. et al. (2018) Linking the International Wheat Genome Sequencing Consortium bread wheat reference genome sequence to wheat genetic and phenomic data. *Genome Biol.*, 19, 1–10. 30115101 10.1186/s13059-018-1491-4 PMC6097284

[31] Wang W., Wang Z., Li X. et al. (2020) SnpHub : an easy-to-set-up web server framework for exploring large-scale genomic variation data in the post-genomic era with applications in wheat. *Gigascience*, 9, 1–8. 10.1093/gigascience/giaa060 PMC7274028 32501478

[32] Rubin C.-J., Zody M.C., Eriksson J. et al. (2010) Whole-genome resequencing reveals loci under selection during chicken domestication. *Nature*, 464, 587–591. 20220755 10.1038/nature08832

[33] Voight B.F., Kudaravalli S., Wen X. et al. (2006) A map of recent positive selection in the human genome. *PLoS Biol.*, 4, 446–458. 10.1371/journal.pbio.0040072 PMC1382018 16494531

[34] Sabeti P.C., Varilly P., Fry B. et al. (2007) Genome-wide detection and characterization of positive selection in human populations. *Nature*, 449, 913–918. 17943131 10.1038/nature06250 PMC2687721

[35] Chen H., Patterson N. and Reich D. (2010) Population differentiation as a test for selective sweeps. *Genome Res.*, 20, 393–402. 20086244 10.1101/gr.100545.109 PMC2840981

